# N-Terminal Protein Binding and Disorder-to-Order Transition by a Synthetic Receptor

**DOI:** 10.1021/acs.biochem.4c00729

**Published:** 2025-02-20

**Authors:** Niamh M. Mockler, Kiefer O. Ramberg, Ronan J. Flood, Peter B. Crowley

**Affiliations:** School of Biological and Chemical Sciences, University of Galway, Galway H91 TK33, Ireland

## Abstract

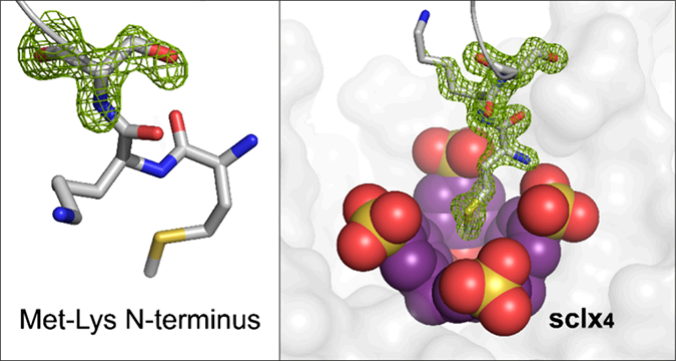

We describe the capture and structuring of disordered N-terminal regions by the macrocycle sulfonato-calix[4]arene (**sclx**_**4**_). Using the trimeric β-propeller *Ralstonia solanacearum* lectin (RSL) as a scaffold, we generated a series of mutants with extended and dynamic N-termini. Three of the mutants feature an N-terminal methionine-lysine motif. The fourth mutant contains the disordered 8-residue N-terminus of Histone 3, a component of the nucleosome. X-ray crystallography and NMR spectroscopy provide evidence for **sclx**_**4**_ binding to the flexible N-terminal regions. Three crystal structures reveal that the calixarene recognizes the N-terminal Met-Lys motif, capturing either residue. We provide crystallographic proof for **sclx**_**4**_ encapsulation of N-terminal methionine. Calixarene capture of intrinsically disordered regions may have applications in regulating protein secondary (and tertiary) structure.

## Introduction

This paper provides evidence for calixarene binding to and structural rearrangement of disordered N-termini in proteins. Specifically, we show that the rigid, bowl-shaped sulfonato-calix[4]arene (**sclx**_**4**_) *captures* cationic N-terminal motifs, resulting in ordered structures. Intercepting disordered proteins with synthetic receptors has broad applications in understanding disease models (e.g., amyloidosis, gene regulation), and supramolecular strategies are well-established.^1−3^ The lysine- or arginine-complexing molecular tweezers can bind a variety of protein features resulting in structural stabilization.^4−7^ Complexation of the intrinsically disordered amyloid-β protein is a case in point where tweezers-induced structural rearrangement can inhibit oligomerization and potentially mitigate disease.^4,6^ All three cationic residues of amyloid-β can bind the tweezers. Sulfonato-calixarenes can also complex amyloid-β, though structural data is lacking.^3^

There have been extensive studies of calixarene complexation of the disordered N-termini of histones.^8−12^ Here, the micro- to nanomolar affinity of **sclx**_**4**_ (and derivatives) for methylated lysines (e.g., trimethylated Lys4 of Histone 3, H3K4me3) has been put to advantage. In addition to providing tools for sensing histone modifications,^8,11,12^ calixarene complexation of the disordered H3 N-terminus can inhibit binding to partner proteins.^9,10^ N-terminal peptide/protein recognition can also be achieved by the donut-shaped cucurbit[n]urils.^13−19^ For example, N-terminal Phe residues are singly or doubly accommodated within the cavity of cucurbit[7]uril or cucurbit[8]uril, respectively.^13,14^ Recent work has expanded the repertoire of peptide guests for cucurbit[8]uril to include dipeptide motifs at N-terminal or nonterminal positions.^15,16,19,20^ A cocrystal structure of cucurbit[8]uril with the heptapeptide GGLYGGG involves encapsulation of the Leu-Tyr motif, and the formation of a neat secondary structure supported in part by an intramolecular hydrogen bond as well as (water-mediated) interactions with the macrocycle’s carbonyl rim (CSD entry KODWEJ).^20^

This study focuses on the N-terminal Met-Lys motif, a high-affinity binding site for cucurbit[n]uril (n = 6 or 8).^16,17^ Using isothermal titration calorimetry, Urbach and co-workers reported micromolar affinity between cucurbit[8]uril and the MKA tripeptide (*K*_d_ ∼ 3 μM).^16^ We demonstrated micromolar affinity between cucurbit[6]uril and the ubiquitin-like SAMP2, which has a disordered N-terminus containing Met1 and Lys2.^17^ In ^1^H–^15^N HSQC-monitored titrations, the Lys2 resonance underwent an unusually large chemical shift perturbation in the presence of cucurbit[6]uril. Similar NMR data were obtained with the model protein *Ralstonia solanacearum* lectin (RSL) bearing an engineered N-terminal Met-Lys motif (MK-RSL), indicating that this cucurbituril binding site is transferrable across protein targets. Crystallographic analysis of the complex is lacking as attempts to cocrystallize either SAMP2 or MK-RSL with cucurbit[6]uril were thwarted by self-association and ready-crystallization of the macrocycle.^17^ Considering the importance of the N-terminal ammonium group for protein–cucurbituril complexation,^13−16,19^ it was concluded that cucurbit[6]uril encapsulated either the N-terminal methionine or the neighboring lysine.^17^

In this work, we evaluated **sclx**_**4**_ as a host for the N-terminal Met-Lys motif. **sclx**_**4**_ has a similar cavity size to cucurbit[6]uril. The test proteins were MK-RSL variants containing 0, 1, or 2 Ala residues resulting in longer, more flexible N-termini (Table 1). For simplicity we apply a consistent numbering for the N-terminal residues; Met0, Lys1, Ala’, Ala”, Ser2. H3-RSL with an N-terminus bearing the first 8 residues of Histone 3 was also tested. RSL has three lysine residues per monomer (K25, K34 and K83), and each mutant bears one additional lysine at the N-terminus. While RSL has negligible affinity for **sclx**_**4**_ under the conditions used here, RSL mutants bearing the Met-Lys motif form complexes with the calixarene. Both X-ray crystallography and NMR spectroscopy demonstrate calixarene selection and ordering of the N-terminus. H3-RSL appears to have fluxional calixarene binding, as evidenced by NMR spectroscopy. These data support the use of macrocycles as hosts for disordered peptides, with potential applications in regulating protein secondary (and tertiary) structure.

**Table 1:** Test Proteins and Results Summary

**protein name**	**N-terminus**	**sclx**_**4**_**NMR analysis**	**sclx**_**4**_**cocrystals**	**PDB**
RSL	S**SV-**	nonbinding	no	
MK-RSL	MK**SV-**	binding	yes	9GR3
MKA-RSL	MKA**SV-**	binding	yes	9GR4
MKAA-RSL	MKAA**SV-**	binding	yes	9GR5
H3-RSL	ARTKQTAR**SV-**	binding	no	

## Experimental Section

### Materials

Stock solutions of **sclx**_**4**_ (Tokyo Chemical Industry, S0469) were prepared in water and the pH was adjusted to 6.0–7.5. The modified pET25rsl vector encoding MK-RSL was reported previously.^17^ Vectors encoding MKA-RSL, MKAA-RSL and H3-RSL were produced by Genscript. Unlabeled and ^15^N-lysine-labeled RSL and variants were expressed in *E. coli* BL21 as described.^17,21^ Expression of each variant was similar to that of wild type RSL (∼100 mg/L culture), with the exception of MKAA-RSL which had a ∼ 4-fold reduced yield. Proteins were purified by mannose affinity chromatography. The column was equilibrated with 20 mM Tris-HCl, 100 mM NaCl, pH 7.4, and proteins were eluted with the same buffer containing 0.1 M D-fructose. For the purification of H3-RSL, 50 mM MgCl_2_ was included in the buffers to prevent the coelution of *E. coli* proteins. Pure fractions were pooled and concentrated in 20 mM potassium phosphate, 50 mM NaCl and 5 mM D-fructose at pH 6.0 (MK-RSL, MKA-RSL, H3-RSL), or pure water (RSL, MKAA-RSL) via ultrafiltration (Millipore, Amicon Ultra 3 kDa). Protein concentrations were determined spectrophotometrically using ε_280_ = 44.46 mM^–1^ cm^–1^ for the monomer, as described.^17,21^ Mass analysis was performed with an Agilent 6460 Triple Quadrupole LC/MS or Agilent 6530 Accurate-Mass Q-TOF LC/MS (Figure S1 and Table S1).

### Cocrystallization Trials

D-fructose bound RSL and variants were cocrystallized with **sclx**_**4**_ at 20 °C. Typically, 0.5–1.0 mM protein was combined with 1–20 mM **sclx**_**4**_ and 5 mM D-fructose immediately prior to setting up the trials. In trials with H3-RSL, up to 30 mM **sclx**_**4**_ was tested. Sitting drop vapor diffusion experiments were prepared using commercial (JCSG++ HTS, Jena Bioscience) screens applied with an Oryx8 robot (Douglas Instruments). Crystals (Table S2) were reproduced via hanging drop vapor diffusion in 24 well Greiner plates.

### X-ray Data Collection, Processing, and Model Building

Crystals were cryo-protected in the reservoir solution supplemented with 25% glycerol and cryo-cooled in liquid nitrogen. Diffraction data were collected to 1.08 Å resolution with an Eiger X 9M detector at beamline PROXIMA-2A, SOLEIL synchrotron (France). Data were processed using the autoPROC pipeline,^22^ with integration in XDS^23^ followed by scaling and merging in AIMLESS^24^ and POINTLESS^25^ in CCP4. Crystal structures were solved by molecular replacement in PHASER^26^ or MOLREP^27^ using an RSL monomer (PDB 2BT9) as the search model. Coordinates and restraints for **sclx**_**4**_ (T3Y) and D-fructose (BDF) were added to the models in COOT.^28^ Model building in COOT and refinement in PHENIX^29^ were continued iteratively until the electron density and R_free_ could be improved no further. Structures were validated in MolProbity^30^ and deposited in the Protein Data Bank under the codes 9GR3, 9GR4, 9GR5 (Table S3).

### NMR Characterization

^1^H–^15^N HSQC-monitored titrations were performed at 30 °C using a 600 MHz Varian spectrometer equipped with a HCN cold probe, as described.^17,21^ Experiments were performed investigating **sclx**_**4**_ binding to lysine residues of RSL, MK-RSL, MKAA-RSL, H3-RSL. NMR samples comprised 0.1 mM ^15^N-lysine-labeled protein (or 0.05 mM MKAA-RSL) in 20 mM potassium phosphate, 50 mM NaCl, 5 mM D-fructose, 10% D_2_O, pH 6.0. Samples were titrated with 1.5 μL aliquots of 100 mM **sclx**_**4**_, and were adjusted to pH 6.00 ± 0.05 after each addition. Spectra were acquired with 8 scans and 64 increments, with the exception of MKAA-RSL for which 32 scans were required. Spectra were processed in VnmrJ.

## Results

### Protein – **sclx_4_** Cocrystal Structures

RSL is a *C*_3_-symmetric homotrimer with a six-bladed β-propeller fold and two sugar binding sites per monomer. The RSL trimer has a toroidal, tube cake shape with wide and narrow ends.^21,31^ The three N-termini are located in close proximity at the narrow end, and are typically disordered in crystal structures. For example, the extended dicationic N-termini of MK-RSL are disordered e.g. PDB 8C9Y.^31^ In this work, we applied **sclx**_**4**_ to capture disordered N-terminal regions. Mass analysis confirmed that the N-terminal methionine is present in MK-RSL,^17^ MKA-RSL and MKAA-RSL, while it is cleaved in H3-RSL (Figure S1 and Table S1).

**Figure 1:**
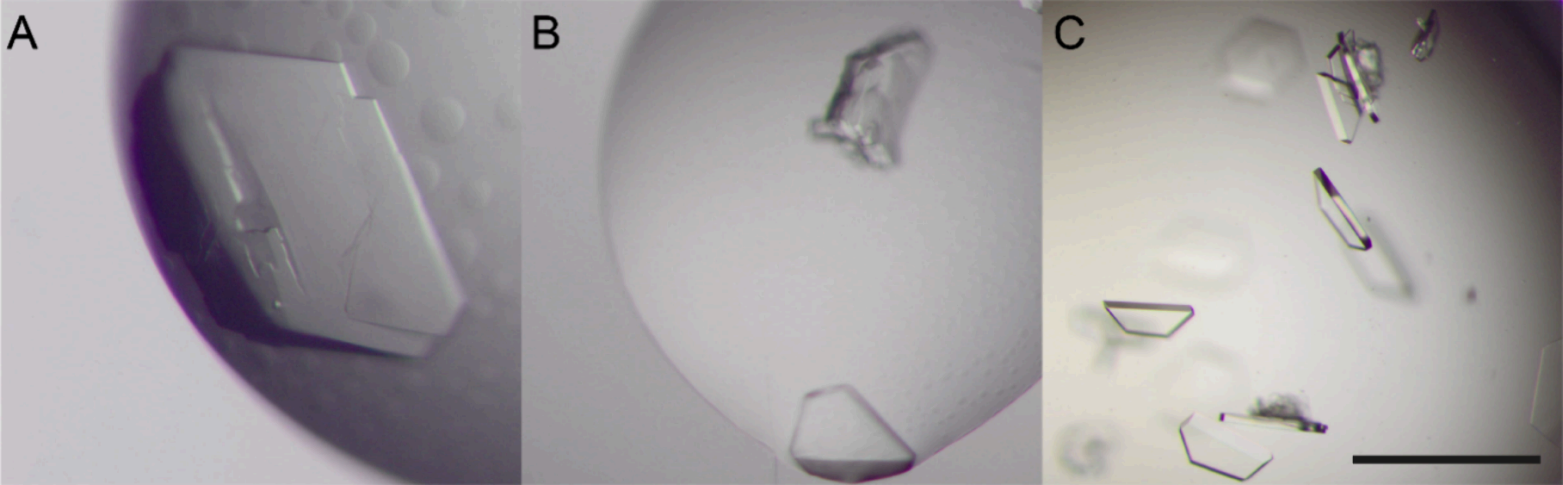
Representative cocrystals of **sclx**_**4**_ with (A) MK-RSL, (B) MKA-RSL, and (C) MKAA-RSL obtained in 1 M diammonium hydrogen phosphate and 0.1 M sodium acetate at pH 4.5. The scale bar is 200 μm.

Crystallization trials of RSL with **sclx**_**4**_ did not yield cocrystals, suggesting that the Lys residues do not bind the calixarene. In contrast, cocrystals of **sclx**_**4**_ with MK-RSL, MKA-RSL or MKAA-RSL grew readily at ∼1 mM protein and 1–20 mM calixarene (Figure 1), in several conditions across the JCSG++ HTS screen (Table S2). Condition E8 (100 mM sodium acetate pH 4.5, 1 M diammonium hydrogen phosphate) yielded all three cocrystals. The crystals were reproduced in this condition via hanging drop vapor diffusion and grew to 100–400 μm dimension within 4 days. Trials with H3-RSL and **sclx**_**4**_ did not yield diffraction-quality cocrystals.

**Figure 2:**
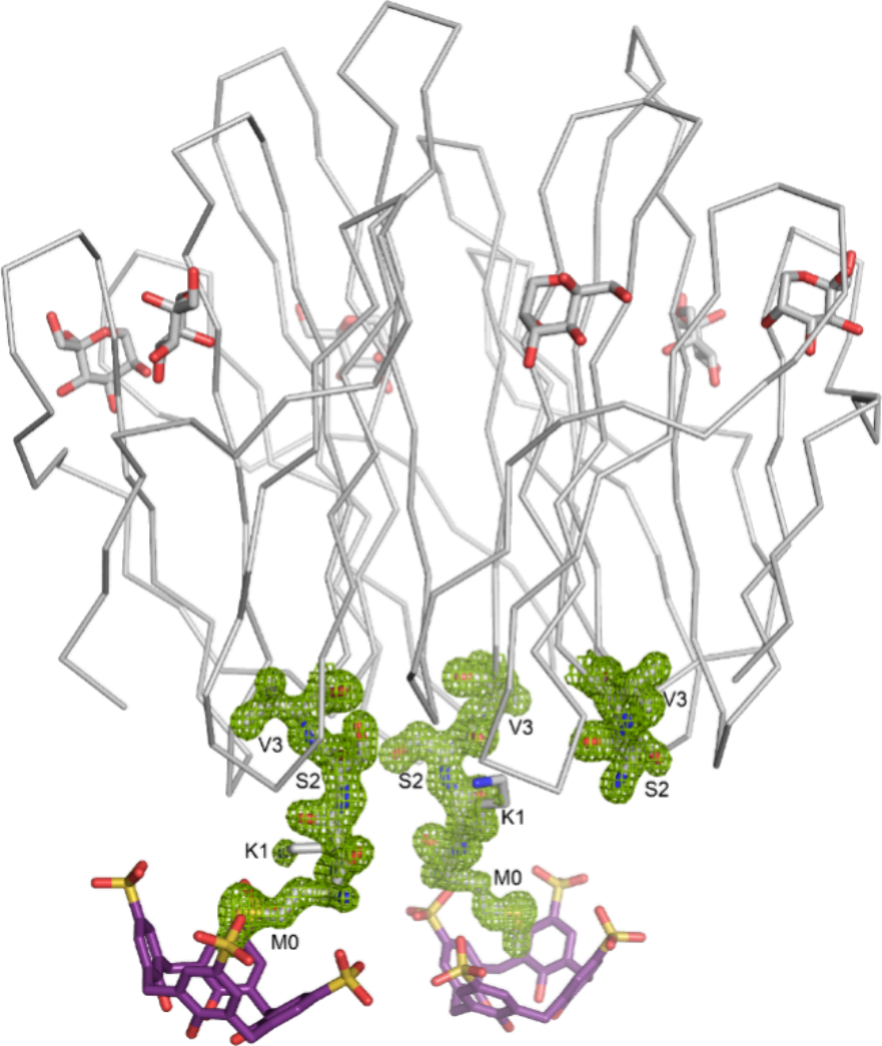
MK-RSL – **sclx**_**4**_ asymmetric unit comprising one protein trimer, two calixarenes, and six β-d-fructose. The refined 2Fo-Fc electron density map (contoured at 1 σ, green mesh) reveals calixarene-bound and ordered N-termini in two subunits. Electron density for Met0 and Lys1 is completely absent in the third subunit. Protein shown as C^α^ trace with the N-termini as sticks.

**Figure 3:**
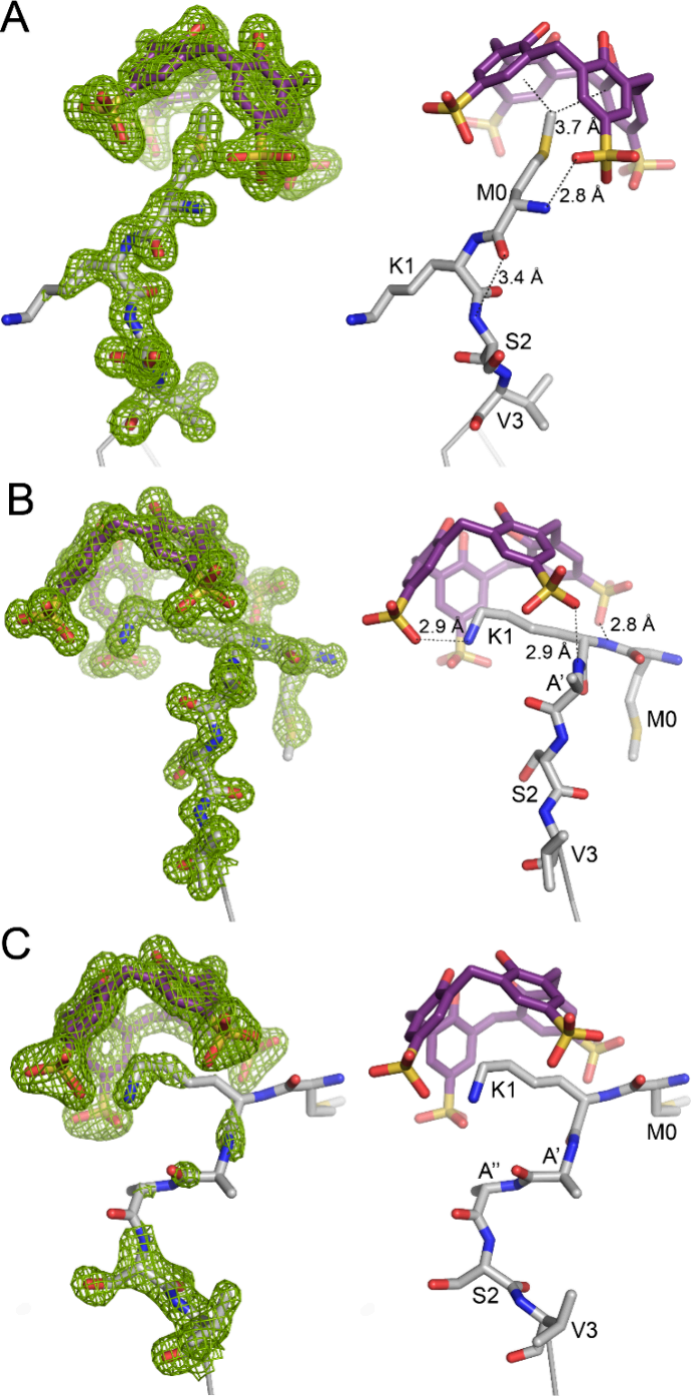
Refined 2Fo-Fc electron density maps (contoured at 1 σ, green mesh) showing **sclx**_**4**_ encapsulation of N-terminal methionine in (A) MK-RSL or Lys1 in (B) MKA-RSL and (C) MKAA-RSL. Noncovalent bonds indicated by dashed lines are described in the text. Note disorder of Met0 in (C).

Using X-ray diffraction data collected at SOLEIL synchrotron, the MK-RSL – **sclx**_**4**_, MKA-RSL – **sclx**_**4**_ and MKAA-RSL – **sclx**_**4**_ cocrystal structures were all solved in space group *P*21 (Table S3). A survey of the PDB suggests that wild type RSL does not crystallize in this space group. The growth of these crystals requires both the N-terminal motif and the macrocycle. The MK-RSL – **sclx**_**4**_ asymmetric unit comprises one protein trimer and two **sclx**_**4**_. While one Met-Lys motif is disordered, clear electron density is evident for the other two N-termini which are bound by calixarenes (Figure 2). Surprisingly, **sclx**_**4**_ encapsulates Met0 instead of Lys1 (Figure 3A). Previous examples of protein–calix[4]arene cocrystal structures feature lysine or arginine encapsulation by the macrocycle.^32−34^ We now report, **sclx**_**4**_ in complex with methionine, albeit an N-terminal residue which allows for favorable charge–charge interactions. Similar interactions occur at the two bound N-termini, with the Met0 side chain trapped in the calixarene cavity and the N-terminal ammonium forming a salt-bridge to a sulfonate (Figure 3A). Met0 forms CH−π bonds with two or more of the phenol-sulfonates as evidenced by a C^ε^···centroid distance of 3.7 Å (Figure 3A). In addition, a weak hydrogen bond between Met0 (C=O) and Ser2 (NH) may contribute to stabilizing the structure.

**Figure 4:**
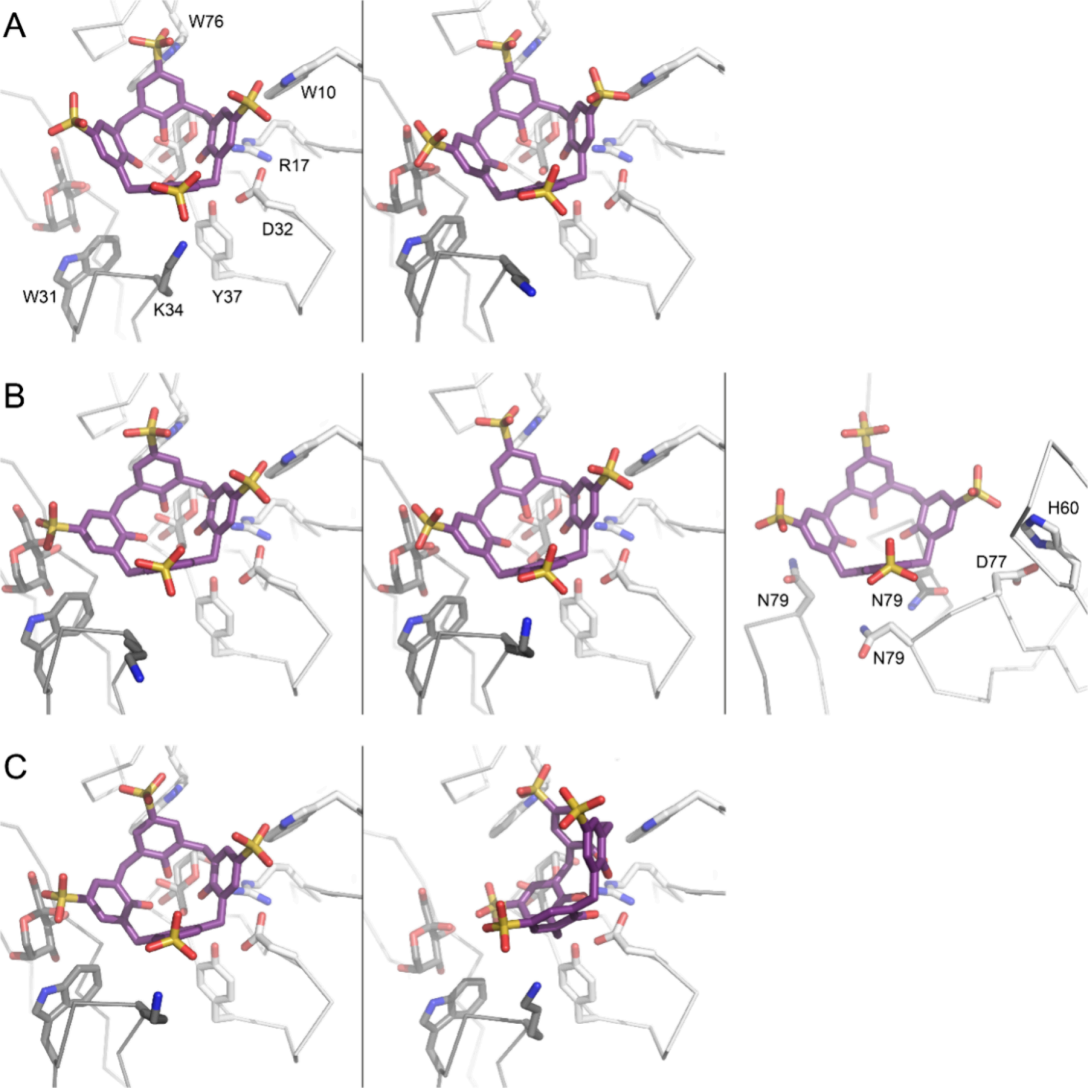
All 7 of the **sclx**_**4**_–protein *exo*-interactions in the (A) MK-RSL, (B) MKA-RSL, and (C) MKAA-RSL cocrystal structures. The calixarene sits in a groove between two or three protein trimers. Different protein trimers are distinguished in greyscale. Note the **sclx**_**4**_ fluxionality in (C). See also Figure S3. Interacting side chains and β-d-fructose shown as sticks. The N-termini are not shown.

In the MKA-RSL – **sclx**_**4**_ and MKAA-RSL – **sclx**_**4**_ structures, Lys1 is encapsulated by the calixarene in the known binding mode, involving at least one salt-bridge and one cation−π bond (Figure 3B, 3C).^32,34^ Elongating the N-terminus results in greater steric accessibility of the lysine, which is now favored over Met0 for **sclx**_**4**_ binding. The MKAA-RSL – **sclx**_**4**_ asymmetric unit is similar to that of MK-RSL – **sclx**_**4**_, with one protein trimer and two calixarenes binding two of the N-termini. While Lys1 is encapsulated, Met0 is disordered (Figure 3C). The MKA-RSL – **sclx**_**4**_ asymmetric unit differs to the other structures, comprising two protein trimers and three macrocycles. One **sclx**_**4**_-bound N-terminus is completely ordered, with clear electron density for the Met-Lys-Ala extension (Figure 3B). The **sclx**_**4**_ at this site also binds a second N-terminus through *exo* interactions (Figure S2). The other two calixarene-bound N-termini are less well-ordered (and modeled with high temperature factors compared to the structure average) while the calixarene is anchored in position. A similar example is observed in the MKAA-RSL – **sclx**_**4**_ structure. Apparently, *exo*-interactions between the calixarene and the protein are important (Figure 4).

In each cocrystal structure, the protein trimers pack in a sheet assembly mediated mainly by protein–protein interactions. The bowl-shaped calixarenes sit in grooves formed at the junction of two or three protein trimers (Figures 4 and S3). All three structures feature calixarenes slotted between the sugar binding sites of two adjacent trimers, with hydrogen bonds between the **sclx**_**4**_ phenols and the primary alcohol of D-fructose. The *exo*-interactions between **sclx**_**4**_ and the protein groove are similar but nonidentical at equivalent sites in all three structures. While one portion of the interface is conserved, the other side varies as a consequence of the *P*21 crystal packing, i.e. relative displacement of the interfacing proteins (e.g. Figure 4A). In most cases, tryptophan side chains play a role by forming CH−π bonds to phenolic rings of **sclx**_**4**_. In some cases, Lys34 forms an *exo*-interaction with the calixarene via a salt bridge to one sulfonate. While clear electron density suggests that the calixarenes are fixed in position, different binding modes are possible. In the MKAA-RSL – **sclx**_**4**_ structure, the two calixarenes bind at similar sites but in different orientations (Figure 4C). This suggests “fluxionality” of the **sclx**_**4**_ – protein *exo*-interaction, i.e. the calixarene can slot into the groove in one-way or another. Moreover, there are different possible *exo* binding sites. In the MKA-RSL – **sclx**_**4**_ structure, two of the calixarenes are slotted between sugar binding sites while a third calixarene resides at the interface of three trimers, interacting with three Asn79 side chains (Figure 4B). Overall, the **sclx**_**4**_*exo*-interactions with the protein appear to be important, harboring the calixarene such that its cavity is exposed and available to capture the flexible N-termini. Noting the variations in the *exo*-interactions, we suggest that the calixarene may bind at well-defined grooves on different proteins.

Interestingly, the *P*21 packing and *exo*-bound calixarene can accommodate N-termini of 2–4 residues in length (Figure 3). However, the larger and more cationic H3-RSL was not amenable to cocrystallization with **sclx**_**4**_.

### NMR Analysis of **sclx_4_**– N-Terminal Complexation

**Figure 5:**
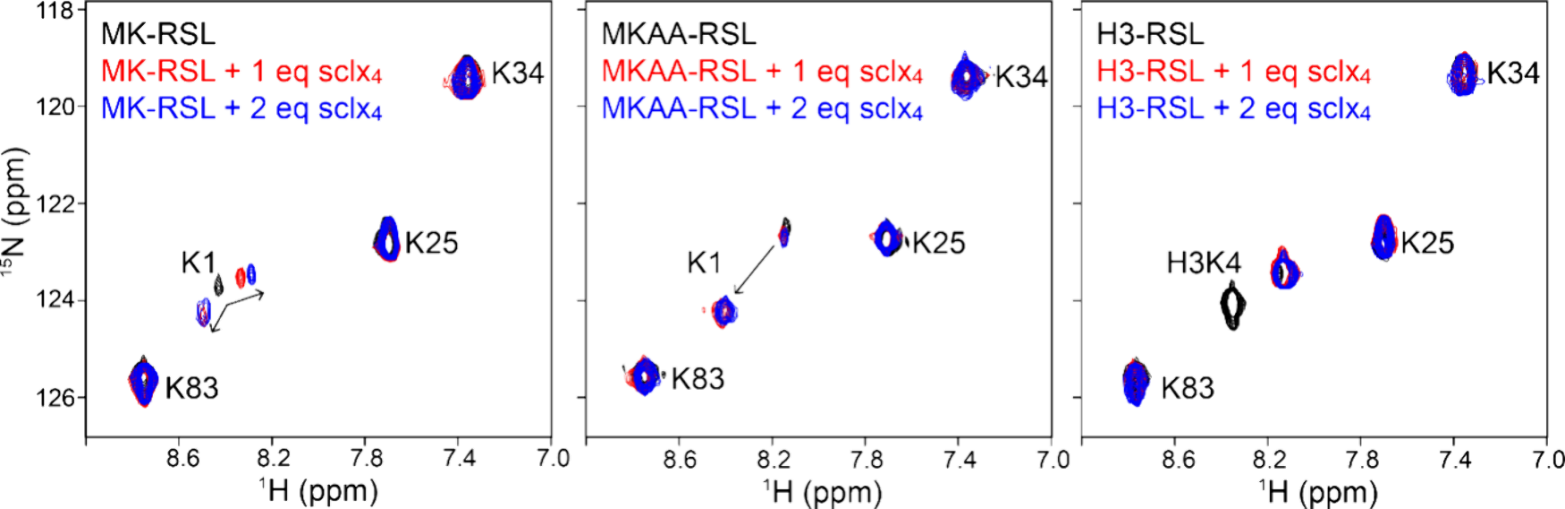
Overlaid ^1^H–^15^N HSQC spectra of ^15^N-lysine-labeled RSL mutants (black contours) in the presence of 1 (red) or 2 (blue) eq **sclx**_**4**_.

Considering the *anchoring* role of **sclx**_**4**_ – protein *exo*-interactions in the crystal structures, further analysis was required to verify that encapsulation of the N-terminus was independent of crystal packing. We conducted ^1^H–^15^N HSQC NMR experiments to study **sclx**_**4**_ – protein interactions in solution. ^15^N-lysine-labeled RSL, MK-RSL, MKAA-RSL and H3-RSL were titrated with the calixarene, allowing for selective analysis of all four lysines in each variant. Consistent with the lack of cocrystals, wild type RSL and **sclx**_**4**_ had negligible interactions in solution. The ^1^H–^15^N HSQC spectrum of ^15^N-lysine-labeled RSL was unchanged by titration with 2 mol equiv (eq) **sclx**_**4**_ in 20 mM potassium phosphate, 50 mM NaCl at pH 6.0 (Figure S4). Thus, **sclx**_**4**_ does not bind to any of the RSL lysines. Under the same conditions, **sclx**_**4**_ complexation at the N-termini of MK-RSL, MKAA-RSL and H3-RSL was evident from NMR experiments (Figure 5). Titration of each variant with **sclx**_**4**_ revealed that the macrocycle binds the extended N-terminus, while the other three lysines were unaffected. The ^1^H–^15^N HSQC spectrum of ^15^N-lysine-labeled MK-RSL has a weak signal for Lys1. Titration with 1 eq **sclx**_**4**_ resulted in splitting of the Lys1 resonance. The upfield shifted component was in fast-exchange while the downfield shifted component was in slow-exchange on the NMR time scale. The fast-exchanging component was further upfield shifted at 4 eq **sclx**_**4**_ (data not shown). These two signals likely correspond to two different forms, for example, **sclx**_**4**_ binding at either Met0 or Lys1. The MKAA-RSL spectrum also has a weak signal for Lys1. At 1 eq **sclx**_**4**_, the original weak signal is unperturbed and a new high intensity signal appears downfield (Figure 5). No further changes occurred at 2 eq **sclx**_**4**_. This system was in slow exchange, suggesting micromolar affinity,^35^ and likely corresponds to lysine capture, as observed in the crystal structure (Figure 3C).

H3-RSL was used to test **sclx**_**4**_ recognition of the cationic and disordered Histone 3 N-terminal motif. While cocrystals of H3-RSL and **sclx**_**4**_ were not obtained, binding was evident in solution. The ^1^H–^15^N HSQC spectrum of ^15^N-lysine-labeled H3-RSL has two signals for the H3K4 residue. One of these signals is weak (in the noise) and the other is intense. Adding 1 eq **sclx**_**4**_ results in a single intense resonance, suggesting interaction in solution. The resonance is unaffected by increasing **sclx**_**4**_ to 2 eq (slow exchange, Figure 5). Although the three potential binding sites (H3R2, H3K4, H3R8) cannot be distinguished, it is plausible that one peptide conformation is stabilized upon interaction with the macrocycle. Calixarene binding at two or more sites on this N-terminus (fluxional binding) may impede cocrystallization. Overall, the slow exchange effects^35^ suggest that the N-termini of MK-RSL, MKAA-RSL and H3-RSL have micromolar affinity for **sclx**_**4**_.

## Discussion

Complex formation between **sclx**_**n**_ and folded proteins is well-studied.^21,31−34,36^ The **sclx**_**4**_ cavity usually encapsulates a single side chain, while the larger, flexible **sclx**_**6**_ and **sclx**_**8**_ accommodate two or more residues on the protein surface.^21,31−34,36^ We have also shown that **sclx**_**6**_ can bind a partially unfolded form of cytochrome *c*.^36^ Here, we demonstrate that **sclx**_**4**_, locked in the bowl conformation, is an effective receptor for disordered N-termini. While **sclx**_**4**_ does not bind wild type RSL, the presence of the N-terminal Met-Lys motif enables complexation. The fixed calixarene conformation is likely advantageous, providing a permanent cavity to trap dynamic residues. **sclx**_**4**_ is comparable to the rigid cucurbit[n]urils, which also offer reliable size-dependent residue recognition.^13−20^ While cucurbit[8]uril can accommodate two N-terminal residues,^13,15,16,19^ cucurbit[6]uril has capacity for one. Both cucurbit[8]uril^16^ and cucurbit[6]uril^17^ can recognize the dynamic N-terminal Met-Lys motif in solution, binding with micromolar affinity. Now, crystallographic evidence of **sclx**_**4**_ complexing the Met-Lys motif unexpectedly reveals the calixarene encapsulating either the N-terminal methionine or Lys1, depending on the peptide length (steric accessibility, Figure 3). It is likely that both complexes occur in solution, with two bound states apparent in the NMR data for MK-RSL (Figure 5). Considering N-terminal complexation, small molecule crystal structures of macrocycle–methionine complexes also involve the ammonium group (CSD entries FAPMAL^37^ and SEGYUA^38^).

Urbach and co-workers have demonstrated peptide folding by cucurbit[8]uril encapsulating an intrapeptide Leu-Tyr motif.^20^ In the case of **sclx**_**4**_ capturing the N-terminal Met-Lys motif, at least one weak intrapeptide hydrogen bond is formed as a result (Figure 3). There is also evidence of additional interactions that may contribute to stabilization. For example, in the MK-RSL – **sclx**_**4**_ cocrystal, an intermolecular hydrogen bond is formed between one of the N-termini and a folded region of a neighboring protein (Met0-N^α^···Ser57-CO).

All three cocrystal structures (1.1–1.3 Å resolution) include disordered, unbound and ordered, calixarene-bound N-termini, highlighting the role of the calixarene in peptide structuring (Figure 3). Similar guest capture occurs in another N-terminal-extended RSL variant in complex with cucurbit[7]uril (PDB 7P2I).^18^ In each crystal structure reported in the current work, the calixarenes are harbored in grooves formed at protein–protein interfaces. The **sclx**_**4**_ – protein *exo*-interactions are important, fixing the calixarene in position while the cavity captures an accessible residue in the flexible peptide. Both binding events are apparently necessary for crystal growth. NMR experiments confirm that **sclx**_**4**_ also binds the disordered N-terminal regions in dilute solution (Figure 5). These results suggest applications of calixarenes as hosts for stabilizing disordered regions against folded protein regions.

## Conclusions

The capture of disordered protein regions by **sclx**_**4**_ is demonstrated both in the solid and solution states. Previous protein – **sclx**_**4**_ complexes involved encapsulation of lysine or arginine residues.^32−34^ Now, we provide crystallographic proof of N-terminal methionine capture. The N-terminal Met-Lys motif is a suitable guest for encapsulation by cucurbit[n]uril (n = 6 or 8)^16,17^ or **sclx**_**4**_. This dipeptide feature may be a useful affinity tag^39^ for macrocycles of suitable size, with applications in purification, biosensing and protein structuring. The three crystal structures reported here demonstrate that Met0 or Lys1 can be captured by the calixarene (Figure 3). Future studies will test other N-terminal Met-X (X = bulky residue) features as the binding target. The crystal structures reveal that the calixarene is harbored at folded portions of the protein (Figure 4), while the cavity captures short, disordered N-termini. Such macrocycle-mediated structuring has applications in studying diverse protein assembly mechanisms.^6,9,15^

## Supporting Information Available

ESI+ mass spectra; crystallization conditions; X-ray data statistics; structure diagrams; HSQC spectra (PDF)

## Accession Codes

*Ralstonia solanacearum* lectin **A0A0S4TLR1**.
