# Supplementary material for: N-Terminal Protein Binding and Disorder-to-Order Transition by a Synthetic Receptor
Source: Biochemistry. 2025 Feb 20;64(5):1092–8. doi: 10.1021/acs.biochem.4c00729 (PMC11883740; doi:10.1021/acs.biochem.4c00729)
Supplement: Supplementary file 1 — bi4c00729_si_001.pdf [file bi4c00729_si_001.pdf]

Supporting Information

N-terminal Protein Binding and Disorder-to-Order Transition by a Synthetic Receptor

Niamh M. Mockler,[a] Kiefer O. Ramberg,[a,b] Ronan J. Flood,[a] and Peter B. Crowley[a]*

[a]School of Biological and Chemical Sciences, University of Galway, Galway, H91 TK33, Ireland
[b]Present address: School of Chemical and Biopharmaceutical Sciences, Technological University Dublin, Dublin, D07 EWV4, Ireland

*Correspondence to: peter.crowley@universityofgalway.ie +353 91 49 24 80

Contents

Figure S1	ESI$^+$ mass spectra of MKA-RSL, MKAA-RSL, H3-RSL	S3
Table S1	Predicted and measured masses from ESI+ mass spectra	S4
Table S2	JCSG++ HTS Conditions yielding protein – **sclx$_4$** cocrystals	S5
Table S3	X-ray data collection, processing and refinement statistics	S6
Figure S2	MKA-RSL N-terminus *exo*-interaction with **sclx$_4$**	S7
Figure S3	Crystal packing in **sclx$_4$** cocrystals with MK-RSL, MKA-RSL and MKAA-RSL	S8
Figure S4	^{1}H-^{15}N HSQC spectra of ^{15}N-Lys-labelled RSL titrated with **sclx$_4$**	S9

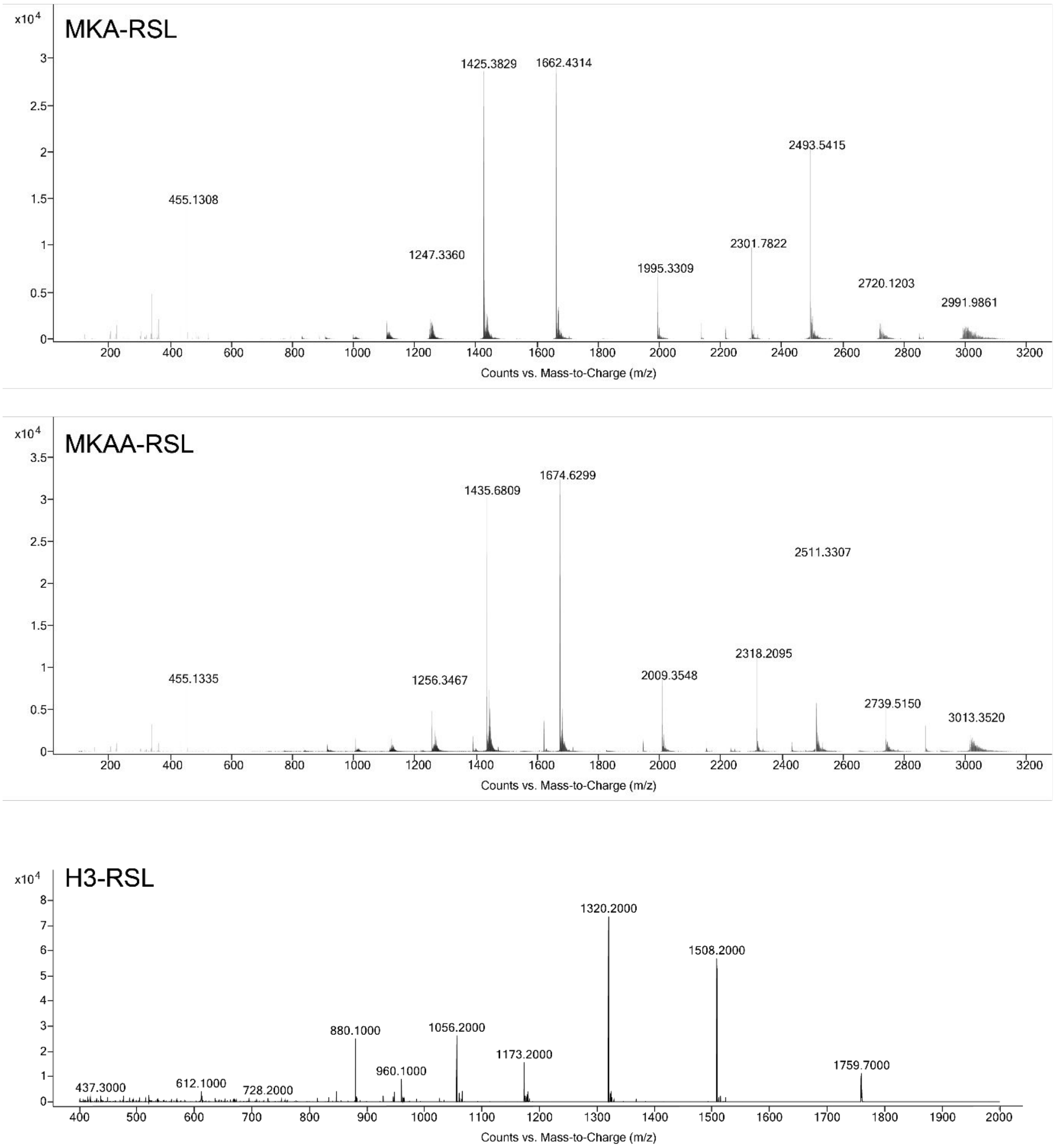

Figure S1: ESI$^+$ mass spectra for MKA-RSL, MKAA-RSL and H3-RSL.

Table S1: Predicted and measured masses from ESI^+ mass spectra (Figure S1).

MKA-RSL monomer			
**m/z**	**charge**	**Molecular Weight (Da)**	**Error (Da)**
1247.34	8+	9970.66	0.73
1425.38	7+	9970.61	0.67
1662.43	6+	9968.53	-1.40
Predicted MW (Da)		9969.99	
Deconvoluted MW (Da)		9969.93	
Standard deviation (Da)		1.21	

MKAA-RSL monomer			
**m/z**	**charge**	**Molecular Weight (Da)**	**Error (Da)**
1256.35	8+	10042.74	0.35
1435.68	7+	10042.71	0.31
1674.63	6+	10041.74	-0.66
Predicted MW (Da)		10041.07	
Deconvoluted MW (Da)		10042.39	
Standard deviation (Da)		0.57	

H3-RSL monomer			
**m/z**	**charge**	**Molecular Weight (Da)**	**Error (Da)**
1320.20	8+	10553.54	1.53
1508.20	7+	10550.35	-1.67
1759.70	6+	10552.16	0.14
Predicted MW (Da)		10552.59	
Deconvoluted MW (Da)		10552.01	
Standard deviation (Da)		1.60	

Table S2: Conditions that yielded protein – **sclx$_4$** cocrystals, as confirmed by X-ray diffraction.

Cocrystal Form	JCSG++ HTS Condition*
MK-RSL – **sclx$_4$**	**B1**: 0.8 M ammonium sulfate, 0.1 M tri-sodium citrate; pH 4.0
	**E8**: 1 M di-ammonium hydrogen phosphate, 0.1 M sodium acetate; pH 4.5
	**G7**: 15% PEG 3350, 0.1 M di-sodium succinate; pH 7.0
MKA-RSL – **sclx$_4$**	**E8**: 1 M di-ammonium hydrogen phosphate, 0.1 M sodium acetate; pH 4.5
	**G8**: 20 % PEG 3350, 0.15 M di-sodium DL-malate; pH 7.0
MKAA-RSL – **sclx$_4$**	**E8**: 1 M di-ammonium hydrogen phosphate, 0.1 M sodium acetate; pH 4.5
	**F7**: 0.8 M di-sodium succinate; pH 7.0

*Condition E8 is common to all three structures.

Table S3. Crystallization conditions, X-ray data collection, processing and refinement statistics for protein – **sclx$_4$** cocrystals.

**Structure**	**MK-RSL – sclx$_4$**	**MKA-RSL – sclx$_4$**	**MKAA-RSL – sclx$_4$**
	**Crystallization**		
**Protein**	1 mM MK-RSL	1 mM MKA-RSL	0.5 mM MKAA-RSL
**D-fructose**	5 mM	5 mM	5 mM
**sclx$_4$**	16 mM	16 mM	5 mM
**Precipitant**	1 M di-ammonium hydrogen phosphate	20 % PEG 3350	0.8 M di-ammonium hydrogen phosphate
**Buffer**	0.1 M sodium acetate pH 4.5	0.15 M di-sodium DL-malate pH 7.0	0.1 M sodium acetate pH 4.5
	**Data collection^a**		
**Light Source**		SOLEIL, PROXIMA-2A	
**Wavelength (Å)**		0.98011	
**Space group**		*P*21	
**Cell constants (Å, °)**	43.678 74.046 43.857 90.00 119.47 90.00	44.048 73.548 75.893 90.00 90.39 90.00	43.725 73.914 43.732 90.00 119.26 90.00
**Resolution (Å)**	38.18-1.19 (1.21-1.19)	75.89-1.08 (1.09-1.08)	38.15-1.32 (1.34-1.32)
**# reflections**	460750 (12689)	1032266 (5234)	383695 (19309)
**# unique reflections**	75076 (3155)	176082 (2849)	54760 (2658)
**Multiplicity**	6.1 (4.0)	5.9 (1.8)	7.0 (7.3)
**I/σ (I)**	13.2 (2.1)	22.1 (2.4)	11.5 (2.1)
**Completeness (%)**	96.9 (81.3)	84.4 (27.4)	96.6 (94.7)
**$R_{meas}{}^b$ (%)**	7.4 (79.1)	4.3 (36.2)	5.6 (102.1)
**$R_{pim}{}^c$ (%)**	2.9 (37.3)	1.7 (24.4)	2.1 (37.6)
**$CC_{1/2}$**	99.8 (64.8)	99.9 (86.3)	99.9 (80.0)
**Solvent content (%)**	38	38	37
	**Refinement**		
**R_{work}**	17.8	16.2	19.8
**R_{free}**	19.9	17.6	22.4
**rmsd bonds (Å)**	0.004	0.004	0.005
**rmsd angles (°)**	0.773	0.772	0.759
	**Composition of asymmetric unit**		
**RSL trimer**	1	2	1
**sclx$_4$**	2	3	2
**water**	346	796	248
**Ave. B-factor (Å^2)**	17.51	14.52	25.38
**Clashscore**	0.71	1.63	2.08
	**Ramachandran analysis,^{d} % residues in**		
**favoured regions**	98.49	96.82	97.41
**allowed regions**	1.51	3.18	2.59
**PDB code**	9GR3	9GR4	9GR5

^{a}Values in parentheses correspond to the highest resolution shell ^{b}Rmeas = ∑hkl √(n/n-1)∑I |Ii(hkl)- ⟨I(hkl)⟩ |/∑hkl ∑i Ii(hkl); ^{c}Rpim = ∑hkl √(1/n-1)∑ n i=1 |Ii(hkl)- |/∑hkl ∑i Ii(hkl); ^{d} ⟨I(hkl)⟩ Calculated in MolProbity.

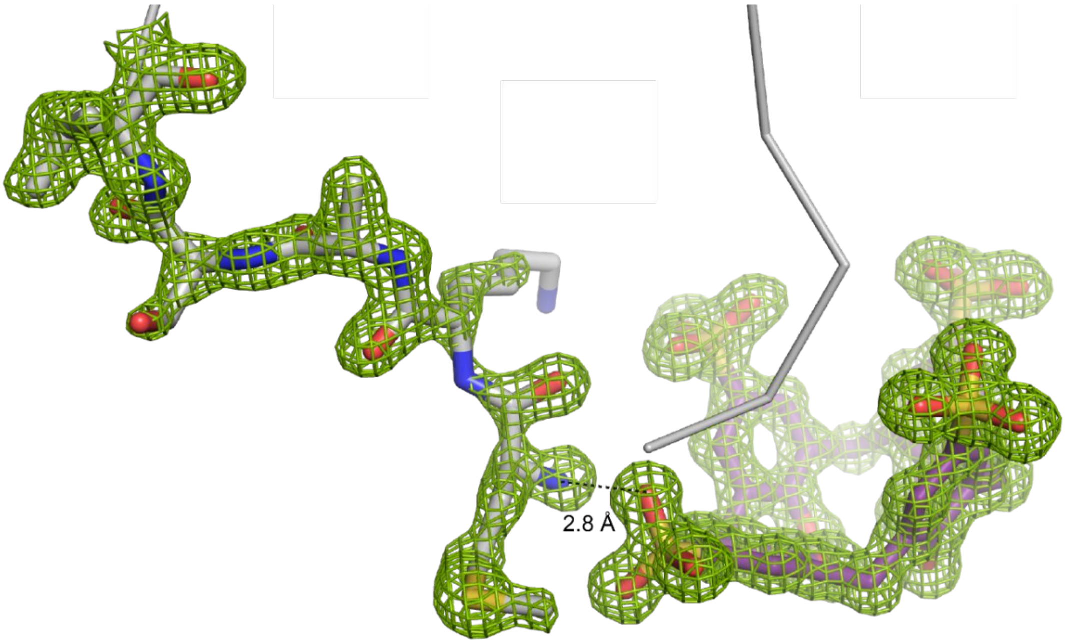

Figure S2. In the MKA-RSL – **sclx$_4$** structure, a calixarene encapsulates one N-terminus (ribbon) and forms an *exo*-interaction with a second N-terminus (sticks) from the same trimer. The dashed line indicates a salt bridge between the N-terminal ammonium and a calixarene sulfonate.

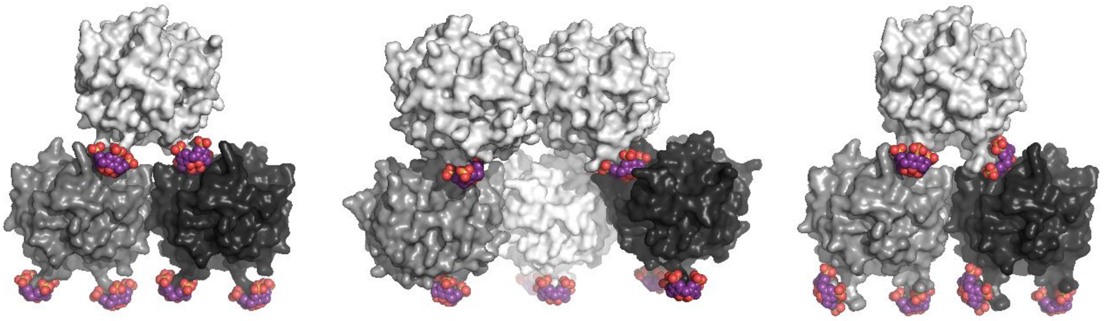

Figure S3: In all three cocrystal structures (space group *P*21), the proteins pack into sheet-like arrangements. Each **sclx$_4$** sits in a groove at junctions of two or more proteins. For clarity, not all symmetry mates (greyscale) are shown.

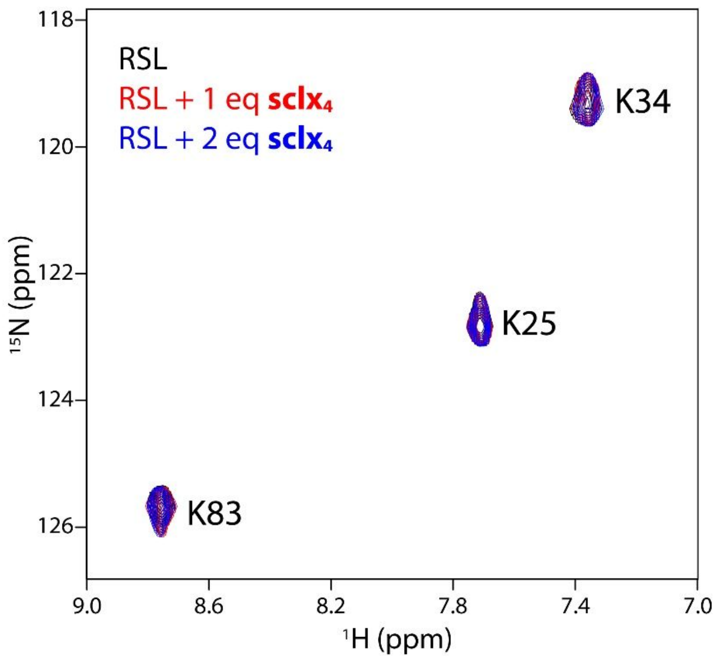

Figure S4. Overlaid ^{1}H-^{15}N HSQC spectra of ^{15}N-lysine-labelled RSL (black contours) in the presence of 1 (red) or 2 (blue) eq **sclx$_4$**.
